# Nitrogen-phosphorus dual-doped auricularia auricula porous carbon as host for Li-S battery

**DOI:** 10.1371/journal.pone.0297677

**Published:** 2024-04-18

**Authors:** Liping Zhao, Ye Zhao, Lihe Zhao, Gang Liu

**Affiliations:** 1 Institute of Chemical and Industrial Bioengineering, Jilin Engineering Normal University, Changchun, PR China; 2 School-enterprise Joint Technology Innovation Laboratory of Novel Molecular Functional Materials of Jilin Province, Changchun, PR China; 3 FAW Tooling Die Manufacturing Co., Ltd., Changchun, PR China; 4 Daqing Oilfield Design Institute Co., Ltd., Daqing, PR China; Universiti Teknologi Petronas: Universiti Teknologi PETRONAS, MALAYSIA

## Abstract

A nitrogen-phosphorus dual-doped porous spore carbon (NP-PSC) positive electrode matrix was prepared using native *auricularia auricula* as solid medium based on the principle of biomass rot. Yeast was introduce and cultured by the *auricularia auricula* solid medium. The freeze-drying and carbonization activation processes made the materials present a three-dimensional porous spore carbon aerogel properties. Yeast fermentation transformed *auricularia auricula* from blocky structure to porous structure and introduced nitrogen-phosphorus dual-doping. The physical and chemical properties of the prepared materials were characterized in detail. Electrochemical performance of NP-PSC in Li-S batteries was systematically investigated. Porous structure and heteroatom-doping improved the electrochemical performance, which is much superior to conventional activated carbon materials.

## Introduction

With the excessive application of fossil fuels and the intensification of the greenhouse effect, research on green and renewable energy is increased more and more. Li-S battery has become a high-profile energy storage and conversion device due to its high energy density and specific capacity. However, three main problems restricte the application of Li-S battery: (1) the conductivity of sulfur is extremely poor [1], with an electronic conductivity of only 5×10^−30^ S cm^-1^ [2]. (2) During the charging-discharging process, the volume effect of the sulfur positive electrode is too obvious [3]. (3) The "shuttle effect" caused by lithium polysulfide (the intermediate product generated during charging-discharging) [4]. Combining with carbon materials or carbon-based-modified materials as matrix can to some extent solve these problems. This is because carbon materials have good structural stability and can accommodate large volume changes [5]. Also, carbon materials with high specific surface area can effectively absorb more lithium sulfide and then alleviate its dissolution in the electrolyte [6]. In short, combining with carbon materials can greatly enhance the performance of sulfur electrode [7].

Biomass reserves are abundant and can be sustainably regenerated, making it an ideal raw material for preparing carbon materials. However, due to the difficult solubility and complex composition, the regulation of biomass carbon morphology and the preparation of ideal composite materials remain a challenge [8]. In recent years, some types of biomass have been reported to be suitable for preparing carbon materials with high specific surface area through direct carbonization activation method [9, 10]. But, for most biomass, it usually has a dense block-like structure, which limits its effective contact with activators and affects its activation effect. If the main internal structure of biomass can be altered and destroyed, it will contribute to the subsequent activation process. The conventional treatment methods include hydrothermal method [11], molten salt method [12] and template method [13]. Nevertheless, these methods require high energy consumption and are not environmentally friendly. So, developing a low-cost and simple method for pre-treatment of biomass is very necessary and meaningful at present.

Additionally, lots of studies have shown that the polarity of the sulfur electrode is not ideal if only using pure carbon materials as the conductive skeleton. Because polysulfide is mainly bound by physical adsorption to inhibit the shuttle effect [14], and sulfur fixation capacity is limited [15]. Moreover, the presence of pure carbon is mostly in the form of sp^3^ hybridization [16], with a low degree of graphitization and relatively poor conductivity [17]. Therefore, finding a simple and mild method to functionalize and modify carbon materials to further improve its conductivity and chemical adsorption capacity for polysulfide is particularly critical. Effectively promote the redox of polysulfide in charge-discharge process is also needed to consider. These are very important for improving the cycle life of Li-S battery, especially the discharge capacity and cycle stability under the condition of high sulfur surface density.

What’s more, it is already demonstrated that heteroatom-doped carbon materials can effectively improve the electrochemical performance of Li-S batteries [18]. This is because heteroatom-doping can increase the force between the oxygen containing functional group and the sulfur atom [19], which can prevent the dissolution of lithium polysulfide in the electrolyte [20], so as to inhibit the shuttle effect [21].

So, a green and inexpensive synthetic approach, using natural *auricularia auricula* and yeast as raw materials was proposed in this work. *Auricularia auricula* is a kind of glial fungus. One of its main chemical components is polysaccharide, and the protein content is also high. The protein contains a variety of amino acids, so *auricularia auricula* is a good carbon and nitrogen source. On the other hand, yeast is a kind of fungus, which can ferment to form spores after absorbing nutrients (such as polysaccharide in *auricularia auricula*). In this paper, the original *auricularia auricula* was employed as the solid medium to culture yeast. After yeast fermentation, the spore carbon could be transformed from bulk to porous material. Yeast spores contain proteins and nucleic acids, which can decompose and convert into N-P heteroatoms during heat treatment. The N-P dual-doped porous *auricularia auricula* spore carbon (NP-PSC) aerogel with unique characteristics was synthesized through simple processes such as freeze drying and annealing. Utilizing yeast fermentation to transform biomass carbon, breaking through the limitation of the original structure on its morphology and structure. The prepared composite material was applied to Li-S batteries and exhibited good electrochemical performance. The characteristic of this work is the "kill two birds with one stone" synthesis strategy. It has achieved the goal of simplifying the preparation process and reducing costs, and has positive practical significance for production applications.

## Materials and methods

### Materials

*Auricularia auricula* was obtained from Paektu Mountain in Jilin Province of China. Yeast was purchased from Changchun Hehe Biochemical Co., Ltd. Potassium hydroxide and anhydrous ethanol were purchased from Aladdin Biochemical Technology Co., Ltd. Anhydrous ethanol was purchased from China National Pharmaceutical Group Chemical Reagent Co., Ltd. Deionized water was self-made. Lithium metal sheet and conductive agent (super-P) were purchased from Wuxi Xinneng Lithium Industry Co., Ltd. Sublimated sulfur (S_8_) was purchased from Aladdin Biochemical Technology Co., Ltd. LiTFSI, 1,3 dioxy cyclopentane (DOL), dimethoxyethane (DME) and lithium nitrate (LiNO_3_) and N-methylpyrrolidone (NMP) were purchased from Aladdin Biochemical Technology Co., Ltd. Button type battery case (CR2032) was purchased from Shenzhen Weifeng Electronics Co., Ltd. Polyvinylidene fluoride (PVDF) was purchased from Solvay Fine Chemicals Co., Ltd. Aluminum foil (Al) was purchased from Beijing Institute of Non Ferrous Metals. Separator (Celgard 2400) was purchased from Celgard Corporation of USA. Lithium metal sheet (Li, 99.99%) was purchased from Tianjin Zhongneng Lithium Industry Co., Ltd. High purity argon (Ar, 99.99%) and nitrogen (N_2_, 99.99%) were purchased form Changchun Zhongsheng Gas Co., Ltd.

### Synthesis of samples

5 g of yeast powder was added into 80 ml of deionized water for dispersion and dissolution, and then 6 g of dried *auricularia auricula* was immersed in the above solution for complete immersion. Yeast absorbed nutrients (such as polysaccharides) from *auricularia auricula* and then undergone anaerobic respiration, decomposed polysaccharides to produce pyruvate, which was fully fermented under anaerobic conditions. After being left for a sufficient period of time, fully fermentation can be achieved. Then *auricularia auricula* was crushed by ultrasonic treatment, a uniform dispersion system was obtained. After freeze-drying and complete removal of water, the sample was calcined in a tubular furnace under nitrogen atmosphere at 800°C for 2 hours. Then the preliminary *auricularia auricula* spore carbon materials was received. Further activation process was required by mixing the above products with KOH powder (KOH/spore carbon weight ratio was set as 3:1), and then the sample was heated under nitrogen flow at 800°C for 2 hours. After KOH activation, the samples was washed several times with 1M HCl solution and distilled water, dried at 60°C. Afterwards, the nitrogen-phosphorus dual-doped porous spore carbon matrix (NP-PSC) was obtained.

Then, NP-PSC and sublimated sulfur (S) was mixed in the mass ratio of 1:3. The mixture was ground with mortar for more than 30 minutes. Appropriate amount of carbon disulfide was dropped during the grinding process to increase the contact between sulfur and composite materials. After that, the ground sample was put into the weighing bottle and covered with tin foil. Then the mixed sample was placed in a vacuum oven at 155°C for thermal diffusion. Finally, the NP-PSC/S positive electrode material was obtained. The synthesis process schematic diagram of NP-PSC/S material is demonstrated as S1 Fig.

### Characterization of samples

The X-ray diffraction (XRD) testing was executed through a diffractometer with the model of X’Pert PRO produced in the Netherlands. The radiation source is Cu Kα, the wavelength is 1.5406 Å, the scanning speed is 10°/min and the scanning angle range is 5 ~ 80°. The X-ray photoelectron spectroscopy (XPS) testing was executed through an Axis Ultra DLD (Kratos) X-ray photoelectron spectrometer. The light source is Al-Kα with a background vacuum of 3×10^−7^ Pa. The scanning electron microscope (SEM) testing was executed by a Hitachi S-4700 instrument produced in Japan, with an acceleration voltage of 25 kV. The instrument for transmission electron microscopy (TEM) testing is FEI Tecnai G2 F30 Transmission electron microscopy. The acceleration voltage is 300 kV. The BET test of the material were tested and analyzed using a nitrogen/desorption instrument produced in the United States (model Micromeritics ASAP 2010). The thermogravimetric (TG) testing was implemented by a SDT Q600 thermogravimetric analyzer produced by TE Company in the United States. The atmosphere used for the test is nitrogen, with a temperature range of room temperature to 600°C.

### Battery assembly and electrochemical performance testing

Electrode fabrication: The mass ratio of active material, conductive agent and binder was set as 7:2:1. A little NMP was added to prepare a black slurry with appropriate viscosity. The adhesive was a mixture of polyvinylidene fluoride (PVDF) and NMP in a mass ratio of 1:10, with a dissolution temperature of 50°C. The conductive agent was acetylene black (super-P). The black slurry obtained can be coated on clean aluminum sheets after 2 hours of magnetic stirring. After dried at 60°C for 6 hours, the electrode was put into a tablet press for pressing, and the pressure was 18 MPa. After drying the completed electrode for 6 hours, the final sulfur-containing positive electrode was obtained.

Battery assembly: The assembly sequence was: positive electrode shell→sulfur positive electrode→separator (two layers)→electrolyte (25 ~ 30 uL)→lithium plate →spring plate→ negative electrode shell. The battery cases were all CR2032 button type. Pure metal lithium plates as the negative electrode and microporous polypropylene as the separator (double layers). The electrolyte was selected as LiN(CF_3_SO_2_) _2_/DOL+DME (volume ratio of 1:1). 4% mass ratio of LiNO_3_ was also added.

Constant current charging-discharging test: After placing the assembled battery for 3 hours, a constant current charging-discharging test was conducted using a LAND CT2001A multi-channel battery detection system. The voltage range was 1.5 ~ 3 V and the current density was varied from 0.1 C to 3 C as needed. The testing conditions were room temperature.

The cyclic voltammetry (CV) and electrochemical impedance test was subjected by the Shanghai Chenhua electrochemical workstation (CHI660C). The scanning rate of CV test was 0.1 mV s^-1^ and the voltage range was 1.5 ~ 3 V. The frequency range of electrochemical impedance test was 0.01 Hz to 100 kHz and the voltage amplitude of was 5 mV.

## Results and discussion

### XRD analysis of materials

**Fig 1:**
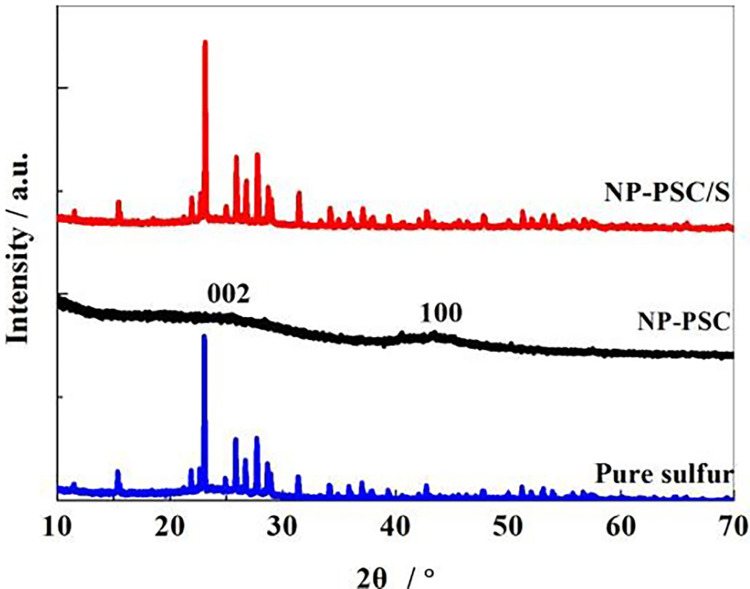
XRD patterns of sulfur, NP-PSC and NP-PSC/S samples.

Fig 1 is the XRD pattern of elemental sulfur, NP-PSC and NP-PSC/S samples. It can be seen that NP-PSC and NP-PSC/S exhibit two distinct peaks at 22.6° and 43.5°, corresponding to the (002) and (100) crystal planes of graphite type carbon, respectively. The peaks both behave broad shape, indicating that the carbon structure is amorphous (the form of amorphous carbon). NP-PSC only owns peaks of carbon and NP-PSC/S does not display any other peaks except for sulfur and carbon peaks, indicating that the materials prepared by this method possess a high purity.

### XPS analysis of materials

**Fig 2:**
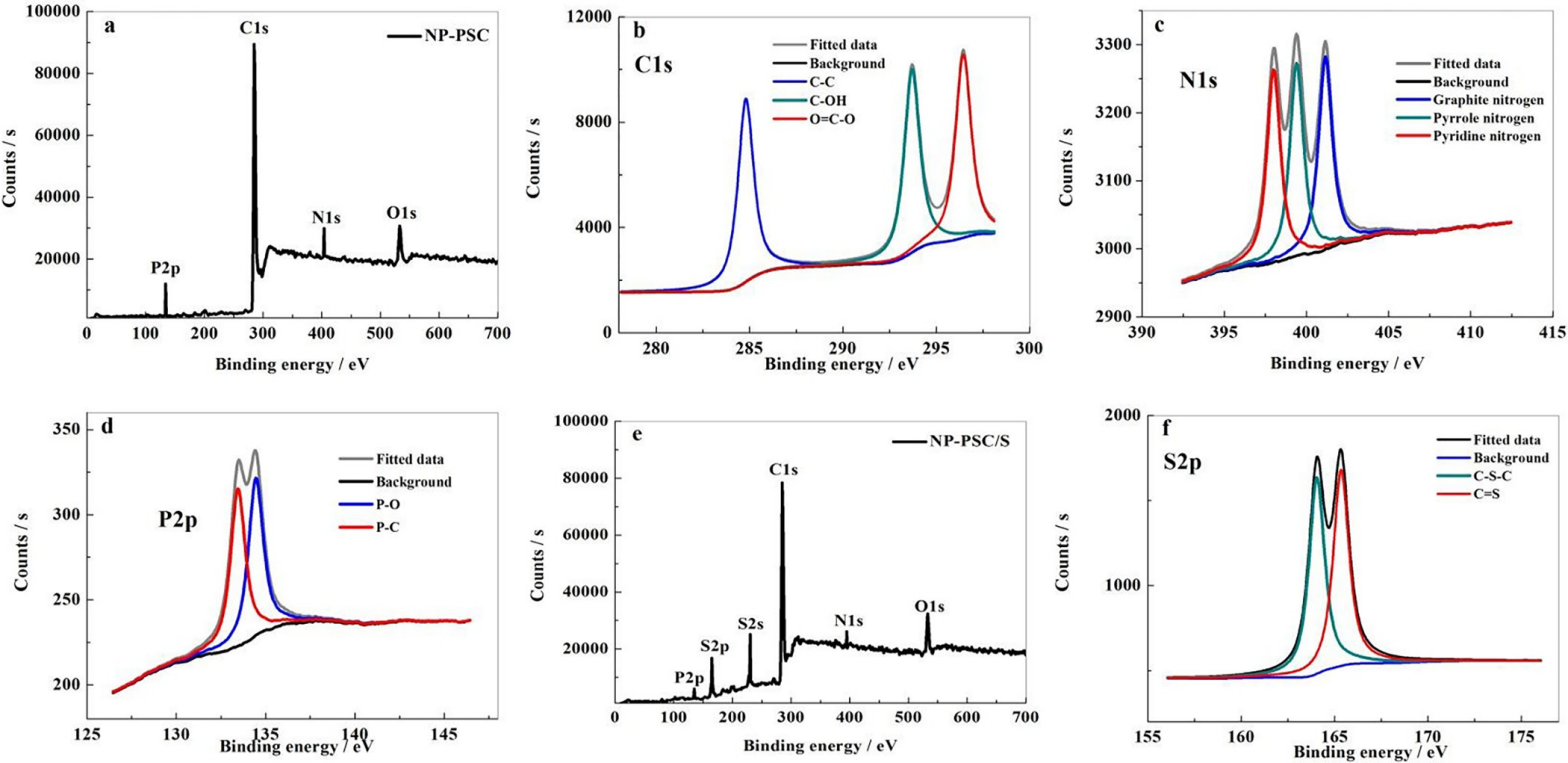
XPS survey (a) and high resolution XPS spectra of Cls (b), N1s (c) and P2p (d) of NP-PSC sample. XPS survey (e) and high resolution XPS spectra of S2p (f) of NP-PSC/S sample.

In order to further explore the existing forms of elements in the NP-PSC matrix, the X-ray photoelectron spectroscopy (XPS) testing was adopted (Fig 2). It is well known that the yeast spores contain proteins and nucleic acids, which can decompose and convert into N-P heteroatoms during heat treatment. In the XPS survey in Fig 2A, NP-PSC sample exhibits four main characteristic peaks at 533, 404, 285 and 134 eV, which is attributed to the O1s, N1s, C1s and P2P peaks, respectively. The C1s spectra (Fig 2B) can be divided into three independent peaks, corresponding to C-C (284.8 eV), C-OH (293.5 eV) and O = C-O (296.4 eV) [22, 23]. Fig 2C displays the N1s spectral line and its fitting diagram of NP-PSC sample. As shown in the figure, nitrogen element behaves three peaks, with the binding energies from low to high being pyridine nitrogen, pyrrole nitrogen and graphite nitrogen, which is corresponding to 398.0 eV, 399.4 eV and 401.1 eV, respectively. Referring to the previous literature report [24–26], pyridine nitrogen can help to inhibit the dissolution of lithium polysulfide. It is because that the carbon atom doped with pyridine nitrogen is positively charged, while the polysulfide ion is negatively charged, so nitrogen-doped carbon materials can effectively absorb lithium polysulfide and reduce its dissolution. For the P2p spectra (Fig 2D), it contains two peaks of 133.45 eV (P-C) and 134.45 eV (P-O) [27]. The dual-doping of N-P heteroatoms in NP-PSC sample can effectively improve the conductivity and increase the active site, which is particularly important for enhancing the electrochemical performance of Li-S batteries [28–30]. The XPS results of NP-PSC/S sample was also displayed in Fig 2E. The XPS survey of NP-PSC/S (Fig 2E) exhibits six main characteristic peaks at 533, 395, 285, 230, 165 and 135 eV, which is attributed to the O1s, N1s, C1s, S2s, S2p and P2p peaks, respectively. From the high resolution spectra of S2p (Fig 2F), we can see that it divides into two independent peaks, corresponding to C-S-C (164.1 eV) and C = S (165.1 eV), respectively. The XPS results demonstrate that nitrogen and phosphorus atoms were successfully doped into the NP-PSC matrix and a strong interaction between carbon substrate and sulfur occurs after combined with the NP-PSC substrate.

### Morphological analysis of materials

**Fig 3:**
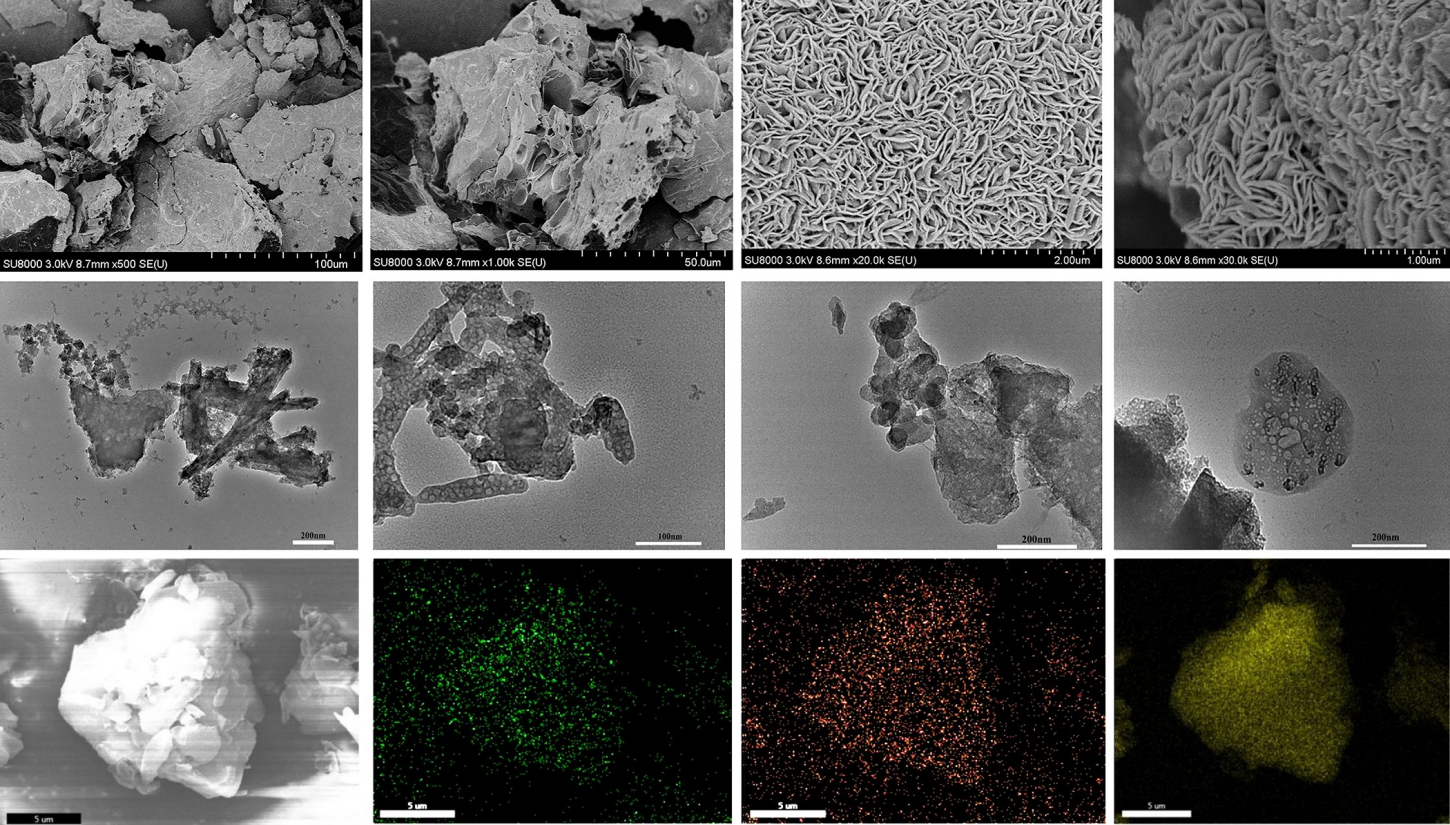
SEM images before fermentation (a ~ b) and after fermentation (c ~ d), TEM images after fermentation (e ~ f) of *auricularia auricula* carbon samples. TEM images of NP-PSC (g ~ h). SEM results (i) and corresponding elemental mappings of N (j), P (k) and S (l) for NP-PSC/S.

The morphology and structure information of samples were explored by SEM (Fig 3A–3D) and TEM (Fig 3E and 3F) method. SEM images before fermentation (Fig 3A and 3B) presents a dense block like structure After fermentation (Fig 3C and 3D), the NP-PSC/S sample is composed of fibrous or scaly aggregates, consisting of layered carbon sheets with gaps. Yeast can absorb nutrients from the *auricularia auricula*, and then reproduce and growth further. Thus, the original blocky and dense structure of *auricularia auricula* (Fig 3A and 3B) was destroyed and then a unconsolidated structure was formed (Fig 3C and 3D). From the TEM results after fermentation (Fig 3E and 3F), it can be seen that the NP-PSC/S composite has an obvious and abundant pore structure, and the pore size is relatively uniform, about a few nanometers (micropores and mesopores). This indicates that the fermentation of yeast owns a good activation effect on the natural *auricularia auricula*. These pore structures contribute to the rapid diffusion of ions and reduce electron transfer resistance. From the morphological analysis, we infer that fermentation make the material structure becoming unconsolidated, and the removal of ice crystals during freeze-drying leads to the formation of layered structures. In addition, due to the organic chemicals in yeast (such as proteins and nucleic acids) contain N-P elements, they can be transformed into carbon materials doped with N-P after heat treatment. The elemental mappings of NP-PSC/S sample was also provided (Fig 3J–3L). The results show that S, N and P elements have been uniformly dispersed and infiltrated into the matrix of NP-PSC/S composite already. The TEM results of NP-PSC sample was exhibited in Fig 3G and 3H. It can be seen that the NP-PSC sample has a porous structure, which is consistent with the BET results.

### BET results analysis of materials

**Fig 4:**
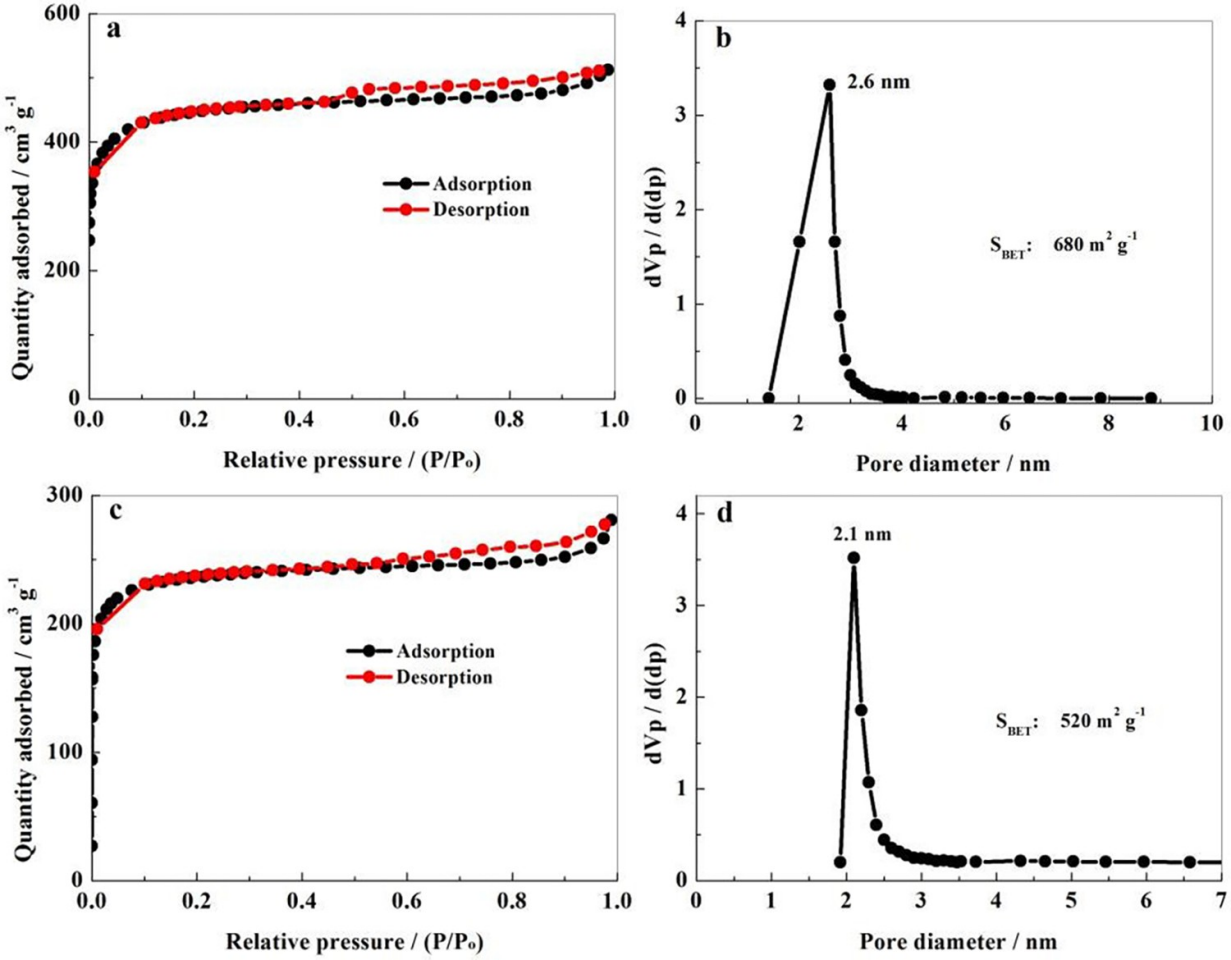
N_2_ adsorption and desorption isotherms (a) and pore size distribution curves (b) of NP-PSC sample. N_2_ adsorption and desorption isotherms (c) and pore size distribution curves (d) of NP-PSC/S sample.

The specific surface area and pore size distribution of NP-PSC matrix was measured through N_2_ adsorption/desorption experiments, as shown in Fig 4. In Fig 4A, the adsorption/desorption equilibrium isotherm can be attributed to a mixture of IUPAC I and IV types. The curves behave an obvious adsorption balance platform. It basically coincides in the area where P/P_0_ is less than 0.45. On the other hand, there is an obvious hysteresis loop caused by capillary condensation in the area of 0.45 ~ 1. From Fig 4B, it can be seen that the specific surface area of the NP-PSC sample is 680 m^2^ g^-1^, belonging to a mesopores-micropores coexisting structure. Obviously, the pore size distribution is relatively uniform, concentrating around 2.6 nm, which is consistent with the TEM results. The results reveal that the NP-PSC matrix possesses a high specific surface area and good porous structure. These porous structures can well accommodate elemental sulfur and withstand a certain degree of volume expansion. For comparison, the BET and pore size distribution results of NP-PSC/S were also provided (Fig 4C and 4D). It can be seen that after loading sulfur, the specific surface area and the pore size of the material both decreases slightly. This is caused by the loading of elemental sulfur into the pore structure.

### Thermogravimetric analysis of materials

**Fig 5:**
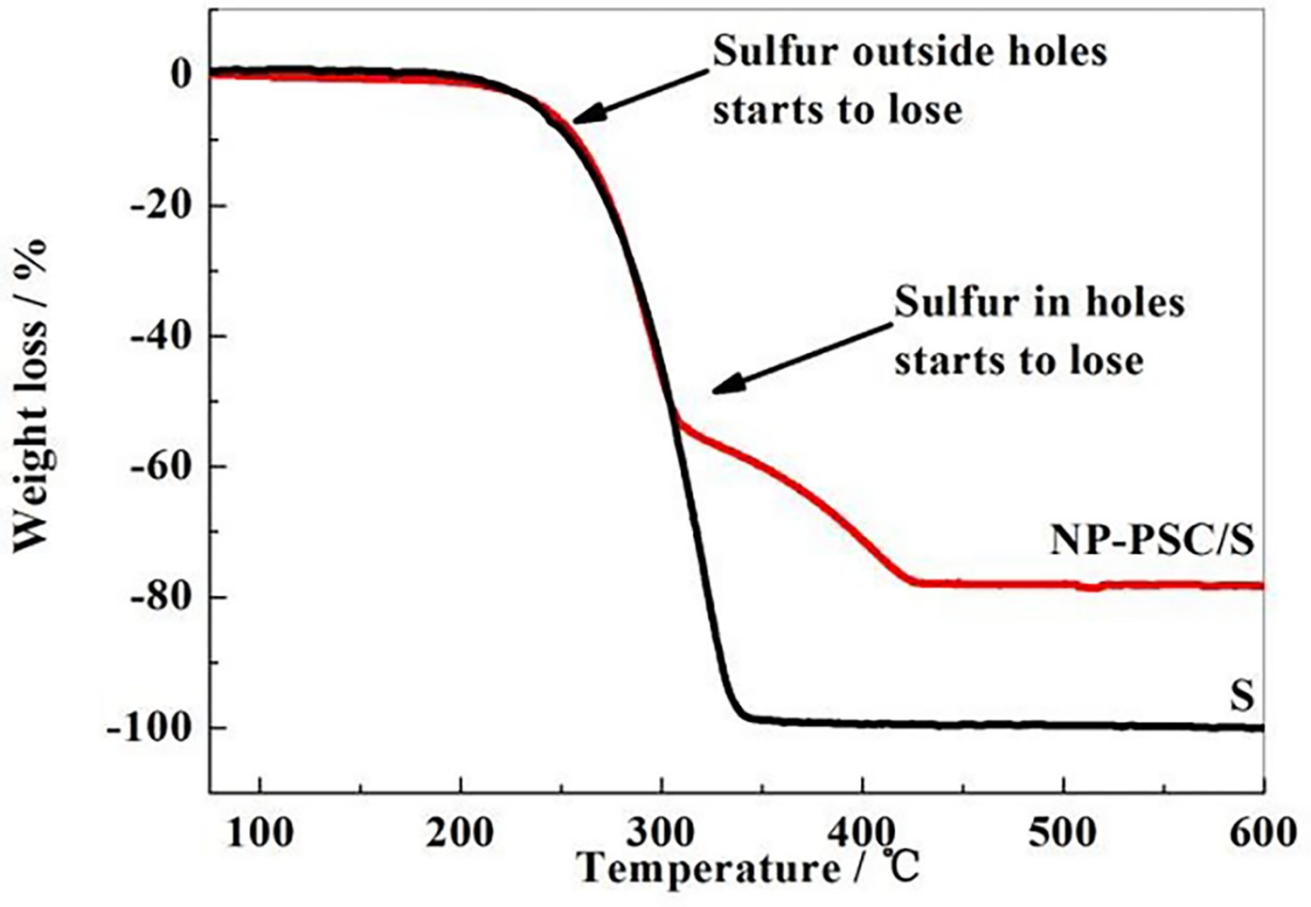
TG curves of NP-PSC/S and elemental sulfur.

The elemental sulfur was compounded with the prepared NP-PSC matrix by hot melt diffusion method, and the weight ratio of sulfur in the NP-PSC/S composite was determined by thermogravimetric analysis. As shown in Fig 5, the curves exhibit two weight loss intervals. The range of 200°C to 300°C corresponds to the loss of sulfur outside the pores, while the range of 300°C to 440°C corresponds to the loss of sulfur inside the pores. Through comparison, it can be concluded that the thermal stability of sulfur in NP-PSC/S is higher than that of elemental sulfur, due to the capillary force of pores in the NP-PSC composite, which can limit the volatilization of sulfur. From the thermal weight loss range, it can be calculated that the weight ratio of sulfur in the NP-PSC/S composite is about 78.2%, indicating that the porous structure of the composite material is conducive to the loading of elemental sulfur.

### Electrochemical performance analysis of batteries

**Fig 6:**
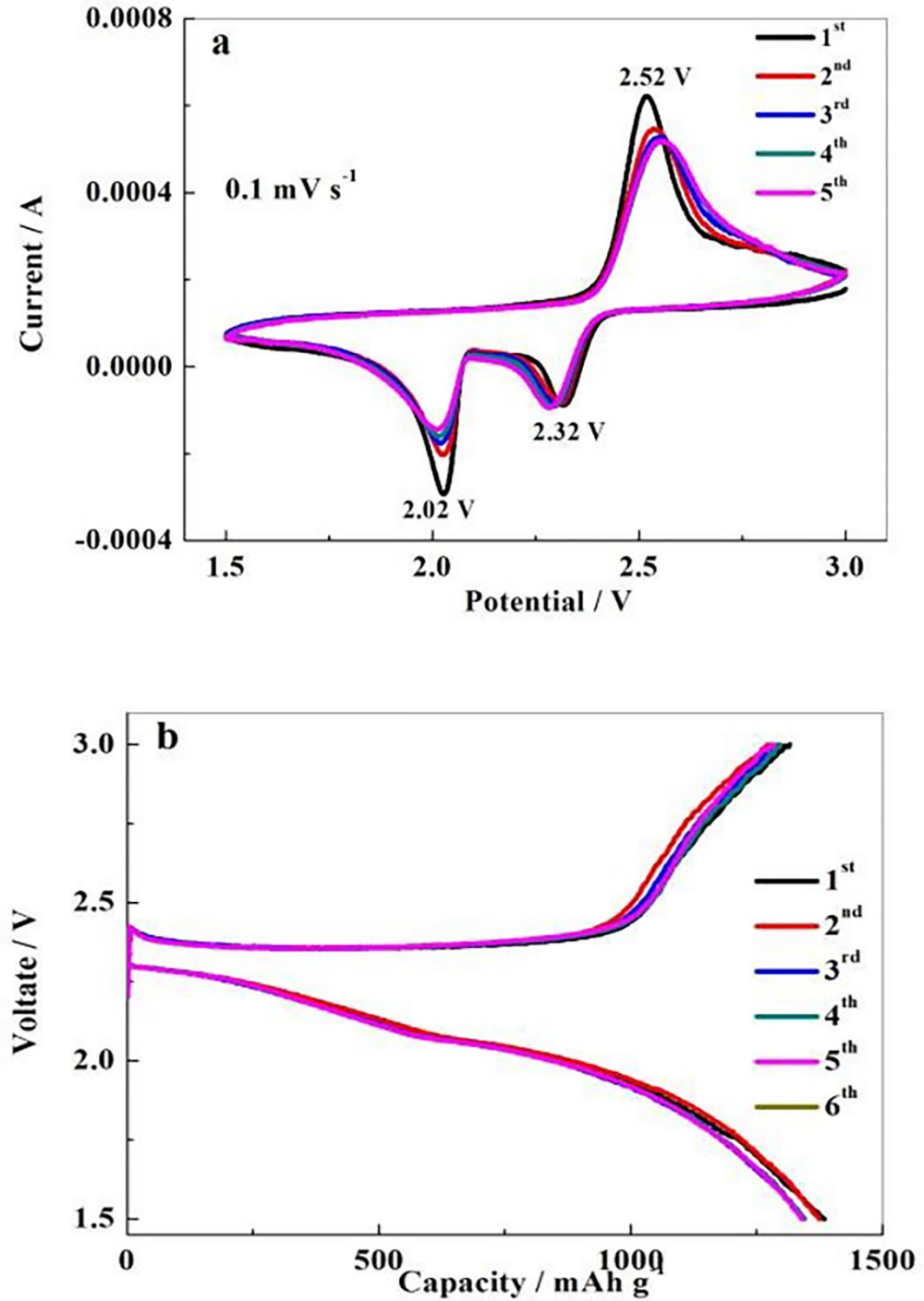
The cyclic voltammograms (a) and charge-discharge profiles (b) of NP-PSC/S electrode.

Fig 6A displays the cyclic voltammetry curve of NP-PSC/S composite. It shows two obvious reduction peaks at 2.32 V and 2.02 V, which respectively corresponds to the formation of long chain lithium polysulfide Li_2_S_8_ intermediate and the reduction process of short chain lithium polysulfide Li_2_S_x_ (x = 4 ~ 6). When it was scanned reversed, an oxidation peak appears near 2.52 V, which corresponds to the formation and final oxidation of polysulfide ions to elemental sulfur. In addition, the peak is narrow shape, and the initial five CV curves can overlap well. This indicates that NP-PSC/S electrode has relatively good electrochemical reversibility and reaction kinetics. CV results prove that NP-PSC/S composite has good conductivity and can effectively inhibit the dissolution of polysulfide. Fig 6B represents the charging-discharging curves of a NP-PSC/S positive electrode during the initial six cycles at a current density of 0.1 C. Apparently, there are two obvious discharge platforms on the discharge curve, which is consistent with the CV results. Moreover, the discharge capacity of the NP-PSC/S electrode is relatively high, with a first discharge capacity of 1385 mAh g^-1^. The coulombic efficiency of the first cycle can reach 96.4% and the cycle stability is relatively ideal.

**Fig 7:**
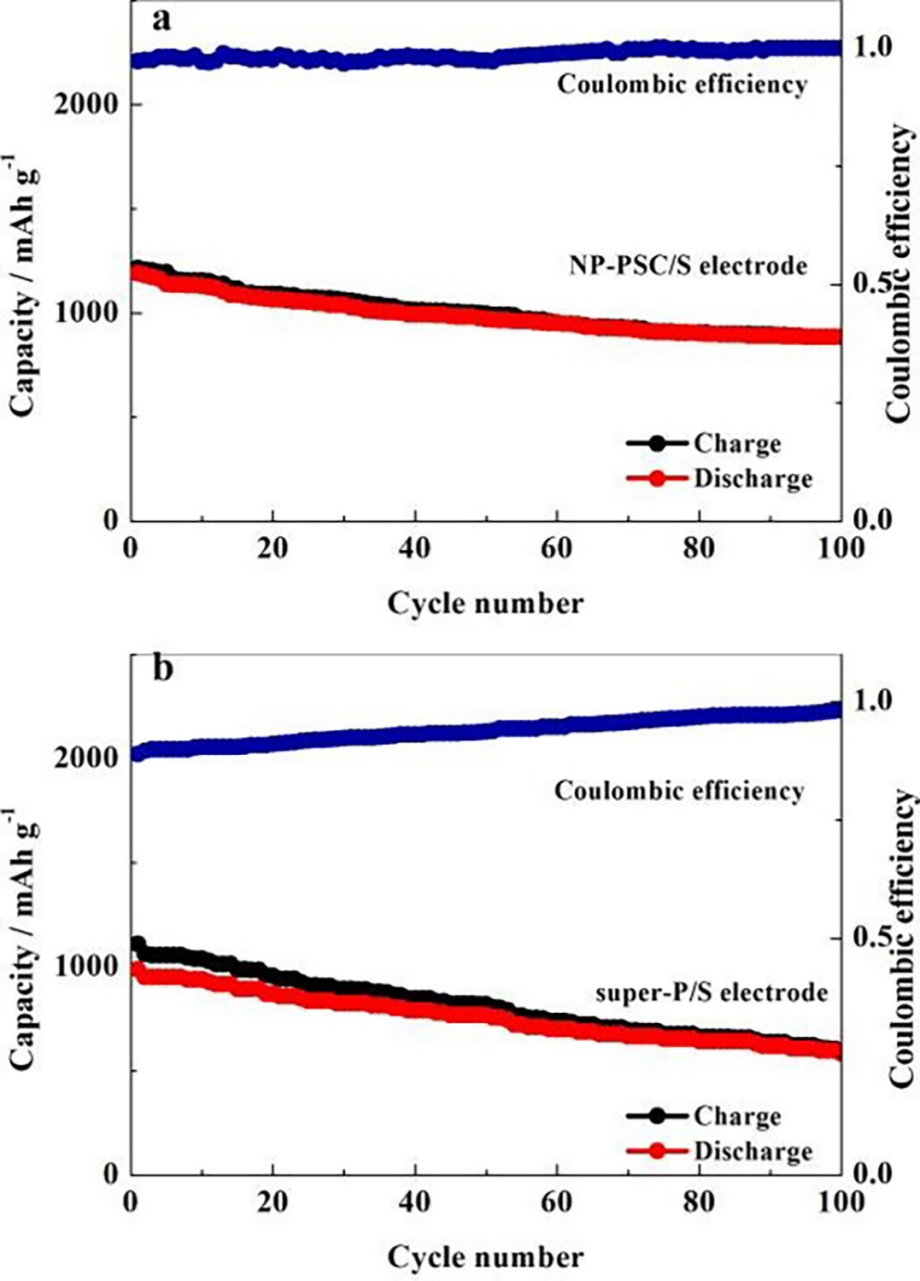
The cycling performance of NP-PSC/S (a) and super-P/S (b) electrodes at 0.2 C.

The cyclic performance and coulombic efficiency curves of NP-PSC/S and super-P/S positive electrodes at a current density of 0.2 C was compared in Fig 7. It can be observed that the first discharge capacity of NP-PSC/S decayed from 1183 mAh g^-1^ to the 100th discharge capacity of 996 mAh g^-1^, while the super-P/S decayed from 987 mAh g^-1^ to 584 mAh g^-1^, with capacity retention rates of 84.2% and 59.2%, respectively. By comparison, it can be concluded that the cycle stability performance of NP-PSC/S is much better than that of super-P/S electrode. This is mainly due to two aspects. One is that nitrogen-phosphorus doping creates a positively charged active site, which can effectively absorb negatively charged polysulfide ions, thus slowing down the dissolution of lithium sulfide in the electrolyte. The second reason is that the porous structure of NP-PSC/S can effectively accommodate sulfur and its compounds, and can also avoid volume changes effectively.

**Fig 8:**
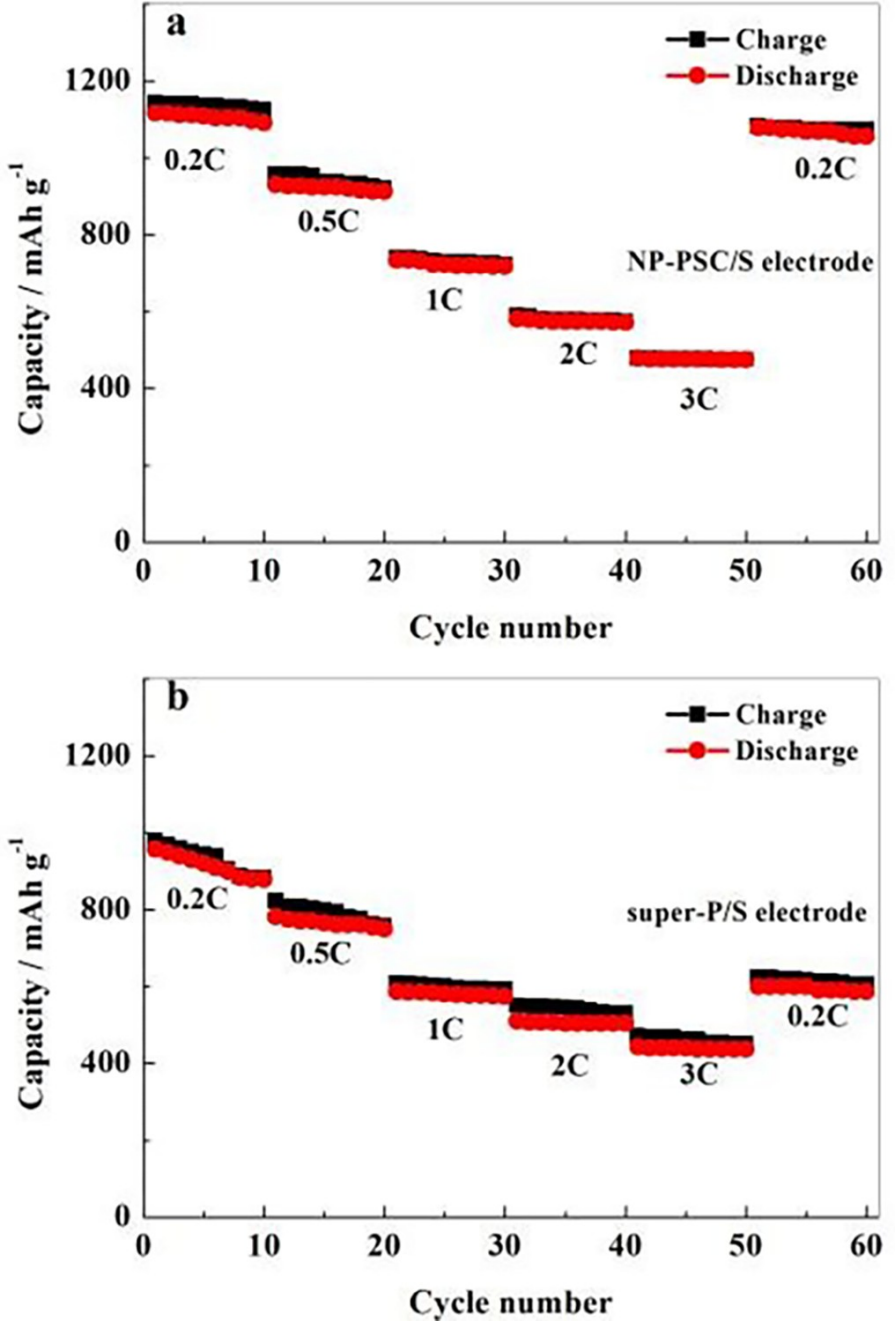
Rate performance of positive electrode NP-PSC/S (a) and super-P/S (b).

The rate performance is also another important indicator for measuring battery performance. Fig 8 shows a comparison of the rate performance using NP-PSC/S and super-P/S as positive electrodes. It is obvious that the NP-PSC/S electrode not only has a higher specific capacity than super-P/S, but also owns significantly better rate performance. When the current density increased from 0.2 C to 0.5 C, 1 C, 2 C and 3 C and then returned to 0.2 C, the capacities of the NP-PSC/S electrode were 1116 mAh g^-1^, 930 mAh g^-1^, 734 mAh g^-1^, 581 mAh g^-1^, 479 mAh g^-1^ and 1078 mAh g^-1^, respectively. After discharging at different current densities, it returned to 0.2 C with a capacity retention rate of over 97%. The NP-PSC/S electrode can still recover to its capacity at low current after high current charging-discharging, indicating its superior high current working ability. The super-P/S electrode bears a lower capacity at high current density. When the current density recovered to 0.2 C, the capacity decreased from 930 mAh g^-1^ to 599 mAh g^-1^, with a capacity loss rate of 35.6%. Surprisingly, compared with biomass materials reported in the published literature [9, 10], the discharge capacity of NP-PSC/S electrode has increased by approximately 10%.

**Fig 9:**
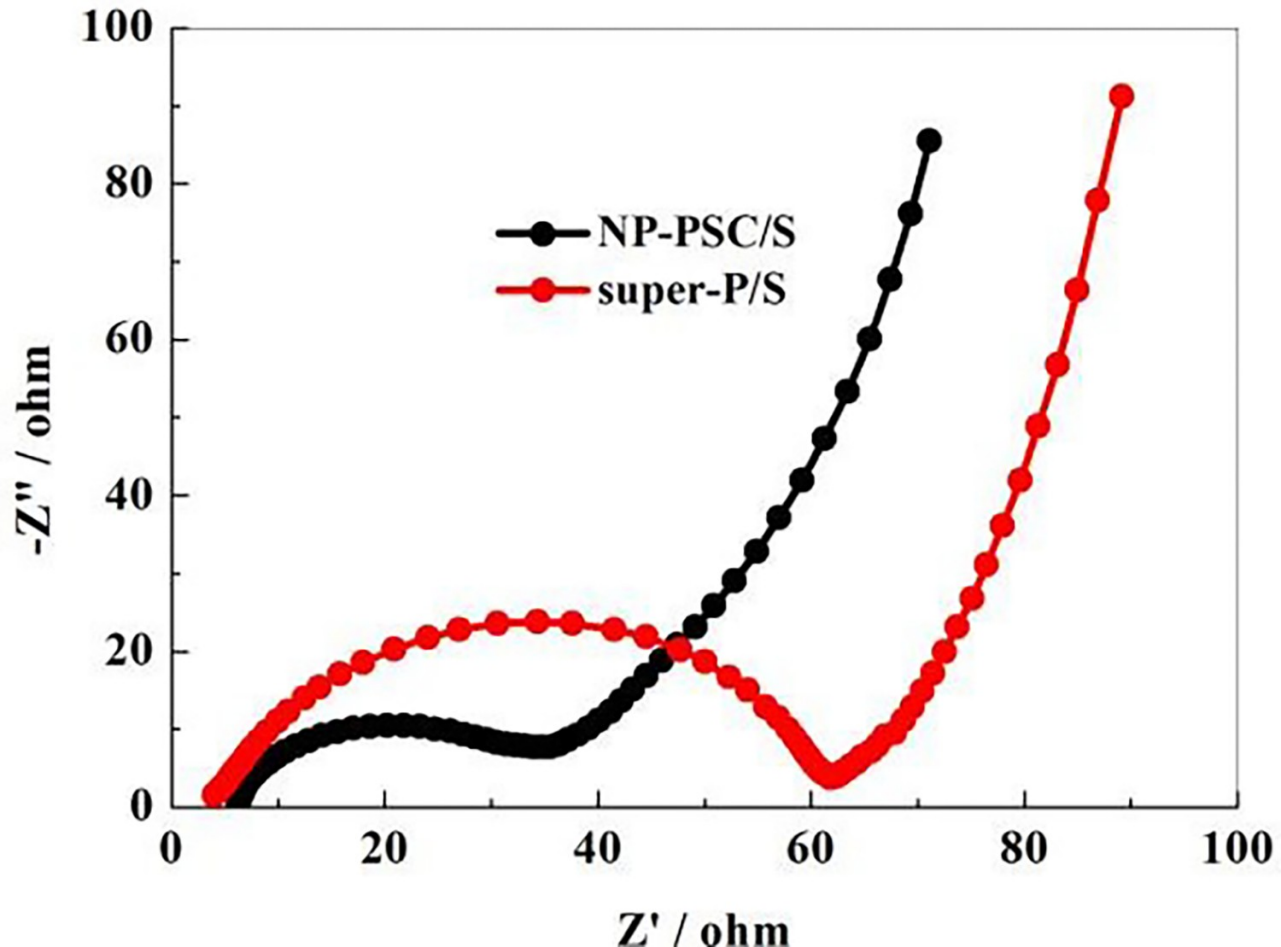
EIS curves of NP-PSC/S and super-P/S electrodes.

Fig 9 is the EIS results of NP-PSC/S and super-P/S electrodes before discharge (100 kHz to 10 mHz). The EIS curves consist of two parts: a semicircle in the high-frequency region (left) and a straight line in the low-frequency region (right). The former corresponds to ohmic impedance and charge transfer impedance, while the latter corresponds to warburg diffusion impedance. The results indicate that the charge transfer resistance and ohmic impedance of NP-PSC/S are both smaller than those of super-P/S. This means that the conductivity of the sulfur significantly increases after combined with the NP-PSC carbon matrix.

## Conclusions

In this work, primary *auricularia auricula* was used as carbon source and solid medium for yeast culturing. The nitrogen-phosphorus dual-doped porous spore carbon (NP-PSC) positive matrix materials was prepared by KOH activating, freezing, drying and calcining processes. The electrochemical performance of NP-PSC/S electrode in Li-S batteries are both superior to conventional activated carbon electrodes. This work innovatively achieved the transformation of the original structure of biomass carbon. It transformed the block carbon to porous carbon and constructed a special structure, which is suitable for activation. The improvement of battery performance is mainly due to the porous structure and nitrogen-phosphorus doping. Porous structures can load more sulfur and alleviate volume expansion. Nitrogen-phosphorus dual-doping can introduce positive active sites. It can effectively absorb negatively charged polysulfide ions, thereby slowing down the dissolution of lithium sulfide in the electrolyte. In summary, this work provides a green-simple method for solving and utilizing biomass rot. Adopting one kind of biomass to activate another to prepare carbon materials with good energy storage characteristics. The method of using biological materials in nature to prepare heteroatom-doped carbon materials not only has the advantages of simple process, low cost, green and environmental protection, but also realizes the comprehensive utilization of waste, which has certain environmental protection and commercial application prospects.

## Supporting information

S1 Fig The synthesis process schematic diagram of NP-PSC/S sample. (DOCX)

## References

[1] Fu ST, Wang HM, Zhong YR, Schaefer S, Li M, Wu MM, et al. High mass loading Li-S batteries catalytically activated by cerium oxide: performance and failure analysis under lean electrolyte conditions. Advanced Materials. 2023, 6, e2302771. doi: 10.1002/adma.202302771 37278254

[2] Yu XM, Wang N, Sun ZP, Shao LY, Shi XY, Cai JJ. Separator modified by a caterpillar-like composite with interconnected N-doped carbon nanotubes decorated Co-MnO heterointerface enabling robust polysulfide adsorption and catalytic conversion for Li-S batteries. Electrochimica Acta. 2023, 457, 142497. doi: 10.1016/j.electacta.2023.142497

[3] Chen M, Liu DX, Meng LC, Zhao Y, Xu JQ, Yin S, et al. Inexhaustible natural celluloses in advanced Li-S batteries: a review. Journal of Materials Chemistry C. 2023, 11 (21), 6819–6833. doi: 10.1039/d3tc01147j

[4] Rayappan PR, Babu MP, Murugan R, Muthuraj D, Ramanujam K. Confined sulfur electrode to achieve quasi-solid state sulfur conversion reaction in Li-S battery. Journal of Energy Storage. 2023, 67, 107601. doi: 10.1016/j.est.2023.107601

[5] Zhao LP, Zhao LH, Zhao Y, Zhang BY, Liu G, Zhang P. Nitrogen-sulfur dual-doped citric-acid porous carbon as host for Li-S batteries. Alexandria Engineering Journal. 2022, 61(7), 5343–5350. doi: 10.1016/j.aej.2021.10.053

[6] Dong TW, Zhang JD, Ai ZY, Ma L, Han B, Jin ZS, et al. Built-in ultrafine CoS_2_ catalysis in confined ordered micro-mesoporous carbon nanoreactors for high-performance Li-S batteries. Journal of Power Sources. 2023, 573, 233136. doi: 10.1016/j.jpowsour.2023.233136

[7] Hao Sun, Xin Li, Chen TQ Xia SX, Yuan T Yang JH, et al. In situ trapping strategy enables a high-loading Ni single-atom catalyst as a separator modifier for a high-performance Li-S battery. ACS Applied Materials & Interfaces. 2023, 15 (15), 19043–19054. doi: 10.1021/acsami.3c02153 37027815

[8] Jing Li, Ji KY Li BY, Xu M, Wang Y, Zhou H, et al. Rechargeable biomass battery for electricity storage/generation and concurrent valuable chemicals production. Angewandte Chemie-International Edition. 2023, 6, e202304852. doi: 10.1002/anie.202304852 37278359

[9] Zhong Y, Deng K, Zheng J, Zhang TT, Liu P, Lv XB, et al. One-step growth of the interconnected carbon nanotubes/graphene hybrids from cuttlebone-derived bi-functional catalyst for lithium-ion batteries. Journal of Materials Science & Technology. 2023, 149, 205–213. doi: 10.1016/j.jmst.2022.11.035

[10] Wu DH, Huang H, Ul Haq M, Zhang L, Feng JJ, Wang AJ, et al. Lignin-derived iron carbide/Mn, N, S-codoped carbon nanotubes as a high-efficiency catalyst for synergistically enhanced oxygen reduction reaction and rechargeable zinc-air battery. Journal of Colloid and Interface Science. 2023, 647, 1–11. doi: 10.1016/j.jcis.2023.05.111 37236099

[11] Li GY, Long YT, Li Z, Li SP, Zheng Y, He BH, et al. Reducing the charging voltage of a Zn-air battery to 1.6 V enabled by redox radical-mediated biomass oxidation. ACS Sustainable Chemistry & Engineering. 2023, 11 (23), 8642–8650. doi: 10.1021/acssuschemeng.3c01799

[12] Zhu ZY, Zhu J, Chen YS, Liu XX, Zhang MC, Yang MX, et al. Leather waste as precursor to prepare bifunctional catalyst for alkaline and neutral zinc-air batteries. Chinese Chemical Letters. 2023, 34 (6), 107756. doi: 10.1016/j.cclet.2022.1077561001–8417

[13] Cui P, Li TZ, Chi X, Yang W, Chen ZH, Han WJ, et al. Bamboo derived N-doped carbon as a bifunctional electrode for high-performance zinc-air batteries. Sustainable Energy & Fuels. 2023, 7 (11), 2717–2726. doi: 10.1039/d3se00315a

[14] Huang YZ, Lin L, Zhang YG, Liu L, Sa B, Lin J, et al. Dual-functional lithiophilic/sulfiphilic binary-metal selenide quantum dots toward high-performance Li-S full batteries. Nano-Micro Letters. 2023, 15 (1), 67. doi: 10.1007/s40820-023-01037-1 36918481 PMC10014643

[15] Li XX, Jia KK, Luo YS, Huang GM, Zhang JW, Zhogn C, et al. In situ composition of Thienothiophene-based covalent organic framework on carbon nanotube as a host for high performance Li-S batteries. Journal of Colloid and Interface Science. 2023, 643, 563–573. doi: 10.1016/j.jcis.2023.03.132 37031070

[16] Zhao LP, Liu G, Zhang P, Sun LQ, Cong LN, Wu T, et al. Nitrogen-sulfur dual-doped porous carbon spheres/sulfur composites for high-performance lithium-sulfur batteries. RSC Advances. 2019, 9 (29), 16571–16577. doi: 10.1039/c9ra00768g 35516355 PMC9064407

[17] Sun R, Qu M, Peng L, Yang WW, Wang ZH, Bai Y, et al. Regulating electrochemical kinetics of CoP by incorporating oxygen on surface for high-performance Li-S batteries. Small. 2023, e2302092. doi: 10.1002/smll.202302092 37292041

[18] Wang W, Wang XY, Shan JW, Yue LG, Shao ZH, Chen L, et al. Atomic-level design rules of metal-cation-doped catalysts: manipulating electron affinity/ionic radius of doped cations for accelerating sulfur redox kinetics in Li-S batteries. Energy & Environmental Science. 2023, 16(6), 2669–2683. doi: 10.1039/d2ee04131f

[19] Jiang ZY, Zhang SL, Dong XB, Xu ZL, Sarwar MT, Yan CY, et al. A diatomite-derived N-doped carbon aerogel with 98% sulfur loading for the enhancement of Li-S battery performance. New Journal of Chemistry. 2023, 47 (10), 4614–4618. doi: 10.1039/d2nj05444b

[20] Dai X, Lv GJ, Wu Z, Wang X, Liu Y, Sun JJ, et al. Flexible hierarchical Co-doped NiS_2_@CNF-CNT electron deficient interlayer with grass-roots structure for Li-S batteries. Advanced Energy Materials. 2023, 13 (21). doi: 10.1002/aenm.202300452

[21] Hu SY, Huang XY, Zhang L, Li GL, Chen SM, Zhang JH, et al. Vacancy-defect topological insulators Bi_2_Te_3_-x embedded in N and B Co-doped 1D carbon nanorods using ionic liquid dopants for kinetics-enhanced Li-S batteries. Advanced Functional Materials. 2023, 5. doi: 10.1002/adfm.202214161

[22] Zhang MD, Mu JW, Li YA, Pan YY, Dong ZL, CHen B, et al. Propelling polysulfide redox by Fe3C-FeN heterostructure@nitrogen-doped carbon framework towards high-efficiency Li-S batteries. Journal of Energy Chemistry. 2023, 78, 105–114. doi: 10.1016/j.jechem.2022.11.026

[23] Zhang W, Li YF, Lv HF, Xie S, Zhu JW, Xu JJ, et al. A comparison study of the electrocatalytic sulfur reduction activity on heteroatom-doped graphene for Li-S battery. Small Structures. 2023, 4 (3). doi: 10.1002/sstr.202200244

[24] Chen L, Zhao CY, Zhao SY, Liu ZY, Lu Y, Bai YX, et al. Facilitating polysulfide confinement using N-doped honeycomb-like carbon with high pyridinic-nitrogen content for high-performance Li-S batteries. Science China-Materials. 2023, 66 (6), 2169–2180. doi: 10.1007/s40843-022-2387-1

[25] Niu JY, Jing DQ, Zhang XH, Su WG, Zhang SC. Nitrogen-doped hollow porous carbon fibers derived from polyacrylonitrile for Li-S Batteries. Carbon. 2023, 206, 435–435.

[26] Choi JM, Saroha R, Kim JS, Jang MR, Cho JS. Porous nanofibers comprising VN nanodots and densified N-doped CNTs as redox-active interlayers for Li-S batteries. Journal of Power Sources. 2023, 559, 232632. doi: 10.1016/j.jpowsour.2023.232632

[27] Li YC, Yan XJ, Zhou ZF, Liu J, Zhang ZH, Guo XS, et al. Synergistic coupling between Fe_7_S_8_-MoS_2_ heterostructure and few layers MoS_2_-embeded N-/P-doping carbon nanocapsule enables superior Li-S battery performances. Applied Surface Science. 2022, 574, 151586. doi: 10.1016/j.apsusc.2021.151586

[28] Zhao LP, Zhao LH, Zhao Y, Liu G. Nitrogen/sulfur dual-doped micro-mesoporous hierarchical porous carbon as host for Li-S batteries. Frontiers in Bioengineering and Biotechnology. 2022, 10, 997622. doi: 10.3389/fbioe.2022.997622 36225606 PMC9548537

[29] Qi S, Peng Q, Xu D, Guo C, Yang J, Dong H, et al. In situ structural evolutions of sulfur-limonene polysulfides encapsulated in yeast-derived porous N-doped carbon spheres for high-performance Li-S batteries. Materials Today Chemistry. 2023, 26, 101138. doi: 10.1016/j.mtchem.2022.101138

[30] Yang JW, Qiao W, Qiao JX, Gao HJ, Li ZX, Wang P, et al. Enhanced performance of Li-S batteries due to synergistic adsorption and catalysis activity within a separation coating made of hybridized BNNSs/N-doping porous carbon fibers. ACS Applied Materials & Interfaces. 2022, 14 (43), 48558–48569. doi: 10.1021/acsami.2c11087 36263683

