# Supplementary figures and images for: Nitrogen-phosphorus dual-doped auricularia auricula porous carbon as host for Li-S battery
Source: PLoS One. 2024 Apr 18;19(4):e0297677. doi: 10.1371/journal.pone.0297677 (PMC11025790; doi:10.1371/journal.pone.0297677)

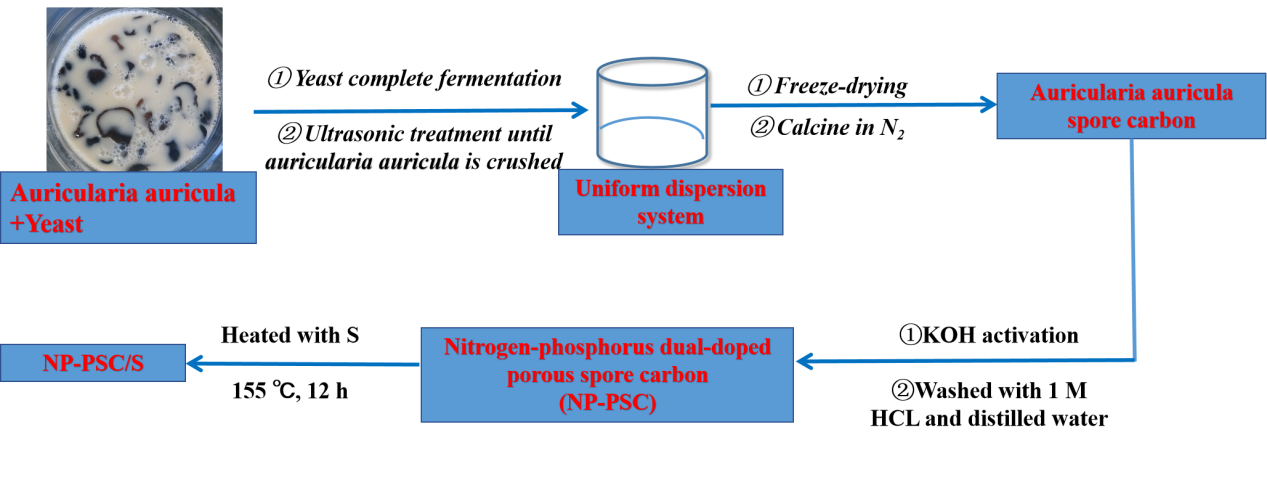
Fig S1. The synthesis process schematic diagram of NP-PSC/S sample.

Supplement: S1 Fig — (DOCX) [file pone.0297677.s001.docx]
